# Activation of the ERK1/2 Molecular Pathways and Its Relation to the Pathogenicity of Human Malignant Tumors

**DOI:** 10.32607/actanaturae.27497

**Published:** 2025

**Authors:** A. G. Emelyanova, M. A. Zolotovskaia, E. V. Poddubskaya, A. A. Modestov, V. S. Prassolov, D. V. Kuzmin, A. A. Buzdin

**Affiliations:** Moscow Institute of Physics and Technology, Dolgoprudny, 141701 Russian Federation; I.M. Sechenov First Moscow State Medical University, Moscow, 119991 Russian Federation; Engelhardt Institute of Molecular Biology, Moscow, 119991 Russian Federation; Shemyakin–Ovchinnikov Institute of Bioorganic Chemistry, Moscow, 117997 Russian Federation; PathoBiology Group, European Organization for Research and Treatment of Cancer (EORTC), 1200 Brussels, Belgium

**Keywords:** ERK1 (MAPK3), ERK2 (MAPK1), gene expression in cancer, ERK molecular pathway activation in oncogenesis, cancer survival biomarkers

## Abstract

Mitogen-activated protein kinases, ERK1/2 (MAPK3/1), play a key role in the regulation of cell growth, differentiation, and apoptosis. We have previously presented evidence proving that activation of the ERK1/2 axis in cancer cells following the administration of therapeutics leads to the overexpression of growth factor receptors and drug resistance. Recently, we have proposed a new bioinformatic technique that enables direct construction of interactome network-based molecular pathways for gene products of interest, as well as quantitation of their activation levels using high-throughput gene expression data. In this study, we, for the first time, algorithmically constructed ERK1/2 molecular pathways and investigated how their activation levels (PALs) affect survival and responsiveness to targeted drugs at the pan-cancer level based on transcriptomic data. We examined a total of 11 287 human tumor profiles from 31 types of cancer, drawn from 53 of our previously published and other literature datasets, looking at patient survival and clinical response to 29 chemo- and targeted therapy regimens. We found that activation of the ERK1/2 pathways has different prognostic significance depending on cancer type. In glioblastoma, sarcoma, lung, kidney, bladder, gastric, colon, and several other cancer types, ERK pathway activation was associated with worse survival. In contrast, the same phenomenon was associated with a better chance of survival in HER2+, luminal A and luminal B breast cancer, and uterine corpus cancer. These trends were consistent with treatment response analysis. At the same time, we found significantly worse associations with the expression levels of individual *MAPK1 *and* MAPK3 *genes: hence, ERK1/2 pathway activation levels can be considered putative biomarkers for predicting clinical outcomes and selecting new personalized treatment strategies, such as the use of MAPK inhibitors.

## INTRODUCTION

Cancer is the second most common cause of death in the world after cardiovascular diseases. According to the World Health Organization (WHO), there were 19.3 million new cases of cancer and 10 million deaths from cancer in 2020, accounting for about 16% of all deaths worldwide [1]. Cancer incidence statistics have been steadily increasing over the past two decades, and cases are projected to rise to 28.4 million in 2040, a 47% increase from 2020 [2]. These trends emphasize the need for increased prevention, early diagnosis, and effective cancer treatment strategies.

Notwithstanding the medical advances in cancer diagnosis and treatment and the availability of targeted therapies, cancer treatment efficacy remains insufficient. It is not uncommon for individual cases of advanced tumors or even entire cancer types to respond poorly to clinically approved chemotherapies and targeted therapies, and, conversely, many cases of individual responses to unlisted drugs or combinations of drugs have been reported [3]. One of the reasons for this is the complexity of the molecular mechanisms of cancer, which makes the development of effective universal treatment strategies a challenge. Therefore, particular attention is being focused on research into the key molecular pathways that regulate the key cellular processes in oncogenesis. In particular, we know that signaling axes such as EGFR, PI3K/AKT/mTOR, RAS/RAF/MEK/ERK, and JAK/STAT play a key role in the regulation of cell growth and division. However, their complex interactions and the presence of parallel signaling pathways make it difficult to develop long-term effective targeted therapeutic regimens. In addition, abnormal regulation of these pathways is often associated with treatment resistance and tumor progression [4, 5, 6].

Of particular interest are the mitogen-activated protein kinases (MAPK) ERK1 and ERK2 (encoded by the *MAPK3 *and *MAPK1 *genes, respectively), which are activated in response to the activation of the RAS-RAF-MEK-ERK signaling axis that plays a key role in tumor cell survival, growth, and proliferation. This axis is closely related to the progression and metastasis of various types of human cancers. Activating mutations in the genes of upstream receptor tyrosine kinases or in the genes encoding the RAS, RAF, MEK and ERK proteins can lead to aberrant activation of ERK1/2 in tumors and, taken together, constitute the most frequent group of mutations in human cancer cells. In general, it is believed that 30–96% of all tumors are characterized by hyperactivation of the RAS/RAF/MEK/ERK signaling axis [7]. ERK1/2, as its downstream component, can be hyperactivated due to uncontrolled activation of receptor tyrosine kinase genes or mutations in the* RAS*, *RAF *and *MEK *genes [8]. Specific inhibitors of the EGFR, BRAF, KRAS, and MEK proteins are included in many standards of anticancer therapy and have proved effective in the therapy of cancers carrying oncogenic mutations in this axis. However, cancer cells often grow resistance to such inhibitors and ERK reactivation is believed to be one of the reasons for such resistance [8, 9].

ERK1/2 proteins play their oncogenic role through abnormal phosphorylation of a wide range of substrates, thereby regulating a variety of tumor-related biological processes, including cell proliferation, differentiation, migration, and angiogenesis [10]. ERK1/2 kinases localize at the crossroads of various signaling pathways, since they are a key node in activating the emergency survival program of tumor cells after the application of receptor tyrosine kinase inhibitors and standard chemotherapy [9].

Hence, these kinases appear to be promising targets for antitumor therapy, in combination with existing antitumor drugs to enhance their efficacy. Therefore, the search for groups of patients in whom the corresponding signaling is elevated may be promising in terms of using specific inhibitors of these MAPK kinases. One approach to identifying such patient groups involves assessing the expression levels of these genes. Variability in gene expression among tumors from different patients has facilitated the adoption of personalized treatment strategies [11]. However, the advent of omics technologies makes it possible to simultaneously examine thousands of genes and other biomarkers [12]. Additionally, various analytical tools allow researchers to summarize results and identify signaling or the biochemical pathways in which the products of these genes are involved, based on data in the literature (functional enrichment). Functional enrichment tools (e.g., overrepresentation analysis and functional class scoring (FCS), commonly used in the analysis of differential gene expression) do not account for the fact that genes operate within complex molecular networks, and their dysregulation in cancer can simultaneously disrupt the regulation of multiple pathways. Different components of a molecular pathway can have distinct functional roles. For example, increased expression of an inhibitory component may suppress the pathway, while activation of a stimulatory component may enhance it. Furthermore, the pathways are often regulated by positive and negative feedback loops, which significantly influence the biological outcomes resulting from pathway activation or inhibition [13, 14]. To address these challenges, a method was proposed to quantitatively assess the activation level of an entire molecular pathway, rather than individual genes, considering the pathway’s architecture and the roles of its constituent components in its activation or suppression. An algorithm was developed for its automatic calculation [14]. The classical algorithm for calculating the molecular pathway activation level (PAL) involves recursive annotation of each node in a given pathway as an activator or repressor, based on the molecular architecture and the nature of each interaction [14]. PAL effectively smooths biases arising from data obtained on different platforms and reduces batch effects [15]. Its values have been used to tell apart normal tissues from tumor ones [16] and predict therapeutic responses in colorectal, renal, and gastric cancer [17, 18, 19, 20]. In addition to the classical PAL calculation, a recently proposed approach constructs the architecture of a molecular pathway as a network of interacting molecules centered around a key node: the central gene. These pathways, referred to as genecentric pathways, are constructed based on the human interactome model and include the maximum number of interactions starting from the central node and leading to every other node in the pathway. PAL of genecentric pathways has demonstrated prognostic and diagnostic value, making it a reliable biomarker for screening, prognosis, and therapy prediction [21, 22].

In this study, we for the first time algorithmically constructed the ERK1 and ERK2 genecentric molecular pathways based on the interactome model previously developed by our group [21]. We sought to investigate the associations of their PALs with survival and responsiveness to targeted drugs at a cancerwide level.

We found that activation of the ERK1/2 pathway has different prognostic values depending on the type of cancer. In glioblastoma, sarcoma, lung, kidney, bladder, gastric, colorectal, and several other cancer types, ERK pathway activation was associated with a worse survival chance. In contrast, it was associated with a better chance of survival in the HER2+, luminal A and luminal B breast cancer, and uterine corpus cancer. These results are consistent with those from the treatment response analysis. In contrast, we found significantly weaker associations with the expression levels of the individual *MAPK1* and *MAPK3 *genes. Hence, the levels of ERK1/2 pathway activation can be considered putative biomarkers for predicting clinical outcomes and selecting new personalized treatment strategies such as the use of MAPK inhibitors.

## EXPERIMENTAL

**RNA expression datasets**

*The Cancer Genome Atlas (TCGA) project dataset. *The RNA sequencing data of solid tumors (STAR counts) and matching normal tissues from the TCGA project were downloaded from the NCI Genomic Data Commons portal [23, 24], along with associated metadata with information on survival, progression, the therapy used, and response to therapy. Only primary tumor samples of cancer types with 100 or more samples were evaluated. In addition, data from the TCGA READ (rectal adenocarcinoma) and COAD (colon adenocarcinoma) projects were combined into the Colorectal Cancer group; similarly, data from the KIRC (renal clear cell cancer) and KIRP (renal papillary cancer) projects were combined into the Renal Cell Carcinoma group. In addition, gliomas and glioblastomas from the TCGA-GBM and TCGA-LGG projects were pooled and reclassified according to the updated WHO classification as shown by Zakharova et al. [25]. The TCGA-BRCA breast cancer dataset was divided into subgroups according to the PAM50 signature [26] due to the high heterogeneity of tumors in this localization. Five molecular subtypes were derived: basal, HER2+, luminal A, luminal B, and normal breast cancer. In the end, a total of 24 cancer types with a total of 8 427 tumor samples were included in the analysis (*Table 1*).

**Table 1:** Statistics for the TCGA RNA expression samples included in the analysis

Cancer type	TCGA project ID	Total number of samples	Number of samples with survival data (OS/PFS)	Number of samples with response to therapy data
Astrocytoma, IDH-mutant. Grade 2	Part of LGG + GBM	110	110/110	22
Basal breast cancer	Part of BRCA	198	198/198	0
Colorectal cancer	COAD + READ	624	619/624	120
Glioblastoma, IDH-wildtype	Part of LGG + GBM	206	204/206	24
HER2+ breast cancer	Part of BRCA	124	124/124	0
Luminal A breast cancer	Part of BRCA	230	229/230	0
Luminal B breast cancer	Part of BRCA	515	514/515	0
Renal cell carcinoma	KIRP + KIRC	823	822/823	15
Urothelial bladder carcinoma	BLCA	406	403/406	92
Cervical squamous cell carcinoma and endocervical adenocarcinoma	CESC	304	304/304	77
Esophageal carcinoma	ESCA	184	184/184	32
Head and neck squamous cell carcinoma	HNSC	520	518/520	77
Hepatocellular carcinoma	LIHC	368	367/368	24
Lung adenocarcinoma	LUAD	516	507/516	98
Lung squamous cell carcinoma	LUSC	501	495/501	61
Pancreatic adenocarcinoma	PAAD	178	178/178	74
Pheochromocytoma and paraganglioma	PCPG	179	179/179	4
Prostate adenocarcinoma	PRAD	497	495/497	40
Sarcoma	SARC	259	259/259	58
Cutaneous melanoma	SKCM	103	103/105	14
Stomach adenocarcinoma	STAD	412	403/412	115
Thyroid cancer	THCA	505	505/505	12
Thymoma	THYM	120	119/120	3
Uterine corpus endometrial carcinoma	UCEC	545	544/545	60

Overall survival (OS) and progression-free survival (PFS) data were assessed in parallel in our analysis. Wherever possible, datasets from the TCGA project were also tapped to analyze the response to therapy according to the RECIST criteria [27] (*Table 1*). For reasons of uniformity and compatibility, the following selection criteria were applied to the TCGA data. First, groups of patients with the same type of therapy, at least 20 patients for each cancer type, were included in the analysis. Second, if the same patient received multiple lines of the same therapy, the best response according to the RECIST criteria was selected for further analysis. Some patients received up to eight lines of therapy, but only the responses to lines 1–3 were included in the analysis, because by the time the later lines of therapy were administered a significant change in the molecular profile of the tumor may have occurred and, therefore, the use of transcriptomic data obtained earlier may be questionable. Finally, only RECIST-defined response groups consisting of at least three patients were considered, for statistical reasons. As a result, data on the response to therapy by patients with 10 cancer types were included (*Table 2*).

**Table 2:** Sufficient TCGA tumor groups with available data on RECIST treatment outcomes

Cancer type	Chemotherapy^1^	Number of patients in the response group^2^
Astrocytoma, IDH-mutant. Grade 2	Temozolomide	SD (n = 12); R (n = 3)
Colorectal cancer	5-Fluorouracil, leucovorin, oxaliplatin	NR (n = 6); R (n = 39)
Glioblastoma, IDH-wildtype	Temozolomide	SD (n = 12); R (n = 4)
Urothelial bladder carcinoma	Cisplatin, gemcitabine	SD (n = 4); R (n = 33); NR (n = 12)
Cervical squamous cell carcinoma and endocervical adenocarcinoma	Cisplatin	R (n = 49); NR (n = 6)
Head and neck squamous cell carcinoma	Cisplatin	R (n = 35); NR (n = 3)
Thyroid cancer	Gemcitabine	SD (n = 4); R (n = 23); NR (n = 25)
Sarcomas	Docetaxel, gemcitabine	R (n = 12); NR (n = 9)
Stomach adenocarcinoma	5-Fluorouracil	R (n = 17); NR (n = 16)
Uterine corpus endometrial carcinoma	Paclitaxel, carboplatin	R (n = 35); NR (n = 5)

^1^Type of chemotherapy used in a patient cohort;

^2^R – responders (total number of patients with RECIST v1.1 Complete Response and Partial Response outcomes);

NR – non-responders (number of patients with RECIST v1.1 Progressive Disease outcome);

SD – patients with Stable Disease outcome according to RECIST v1.1 classification.

*Gene Expression Omnibus (GEO) and Tumor Alterations Relevant for GEnomics-driven Therapy (TARGET) repository datasets. *The datasets included were selected from the previous collection of clinically annotated gene expression datasets with a validated quality of the expression profiles [28]. The solid tumor RNA sequencing data (STAR-counts) from the TARGET project were downloaded from the NCI Genomic Data Commons portal [29]. Microarray gene expression datasets were extracted from the GEO portal [30, 31]. The TARGET-AML dataset (for acute myeloid leukemia) was divided into two sub-datasets based on the presence or absence of busulfan and cyclophosphamide in the treatment regimen. Additionally, the analysis included data from the TCGA project for LGG and UCEC and the combined dataset for lung cancer (LUSC + LUAD) where the information was extracted from the collection by Borisov et al. [28]. A total of 26 additional datasets of nine cancer types, with a total of 2 736 tumor samples, were included (*Table 3*).

**Table 3:** Datasets added from the collection of clinically annotated tumor expression profiles

Cancer type	Dataset ID	Therapy^1^	Number of samples	Number of responder and non-responder patients according to [28]^2^
Breast cancer with different hormonal and HER2 statuses	GSE18728	Docetaxel, capecitabine	61	23R, 38NR
Breast cancer with different hormonal and HER2 statuses	GSE20181	Letrozole	52	37R, 15NR
Breast cancer with different hormonal and HER2 statuses	GSE20194	Paclitaxel, 5-fluorouracil, cyclophosphamide, doxorubicin	52	11R, 41NR
Breast cancer with different hormonal and HER2 statuses	GSE20271	Paclitaxel, 5-fluorouracil, adriamycin, cyclophosphamide	84	18R, 66NR
Breast cancer	GSE22358	Docetaxel, capecitabine	122	116R, 6NR
Breast cancer	GSE23988	Docetaxel, capecitabine	61	20R, 41NR
Breast cancer with different hormonal and HER2 statuses	GSE25066	Neoadjuvant therapy with taxanes and anthracyclines	508	118R, 389NR
Breast cancer	GSE32646	Paclitaxel, 5-fluorouracil, epirubicin cyclophosphamide	115	27R, 88NR
Breast cancer	GSE37946	Trastuzumab	50	27R, 23NR
Multiple myeloma	GSE39754	Vincristine, adriamycin, dexamethasone followed by autologous stem cell transplantation	136	74R, 62NR
Breast cancer with different hormonal and HER2 statuses	GSE41998	Neoadjuvant therapy with doxorubicin, cyclophosphamide, paclitaxel	124	90R, 34NR
Breast cancer	GSE42822	Docetaxel, 5-fluorouracil, epirubicin, cyclophosphamide, capecitabine	91	38R, 53NR
Breast cancer with different hormonal and HER2 statuses	GSE50948	Paclitaxel, doxorubicin, cyclophosphamide, methotrexate, trastuzumab	156	53R, 103NR
Acute myeloid leukemia	GSE5122	Tipifarnib	57	13R, 44NR
Breast cancer	GSE59515	Letrozole	75	51R, 24NR
Multiple myeloma	GSE68871	Bortezomib, thalidomide, dexamethasone.	118	69R, 49NR
Breast cancer	GSE76360	Trastuzumab	48	42R, 6NR
Multiple myeloma	GSE9782	Bortezomib	169	85R, 84NR
Non-small cell lung cancer (lung adenocarcinoma + squamous cell lung cancer + other types)	GSE207422^*^	Anti-PD-1 immunotherapy	24 (8 + 12 + 4)	9R, 15NR
B-cell acute lymphoblastic leukemia	TARGET10	Vincristine sulfate, carboplatin, cyclophosphamide, doxorubicin	98	30R, 68NR
Pediatric acute myeloid leukemia	TARGET20_Busulfan	Polychemotherapy^**^ + Busulfan, cyclophosphamide	54	31R, 23NR
Pediatric acute myeloid leukemia	TARGET20_NoBusulfan	Polychemotherapy^**^	142	62R, 80NR
Williams tumor (nephroblastoma)	TARGET50	Vincristine sulfate, cyclosporine, cytarabine, daunorubicin	122	36R, 86NR
Lung cancer	TCGA_LC	Paclitaxel, optional: cisplatin/carboplatin, rheolysin	35	22R, 13NR
Low-grade glioma	TCGA_LGG	Temozolomide, optionally: mibefradil	131	100R, 31NR
Endometrioid adenocarcinoma	TCGA_UCEC	Paclitaxel, optional: cisplatin/cisplatin, doxorubicin	52	45R, 7NR

^1^Type of chemotherapy, targeted therapy, immunotherapy, or hormone therapy used in a patient cohort.

^2^“R” stands for treatment responders; “NR”, for non-responders.

^*^This dataset was not annotated in [28]. It includes information about patients’ response to immunotherapy according to the RECIST criteria and, therefore, was added to the analysis.

^**^Polychemotherapy regimen included: asparaginase, cytarabine, daunorubicin hydrochloride, etoposide, gemtuzumab ozogamicin, and mitoxantrone hydrochloride.

*Original clinical datasets. *We also included proprietary clinically annotated RNA sequencing datasets previously obtained in our laboratory and published elsewhere. When available, the treatment outcomes were assigned according to the RECIST criteria [27]. The following original datasets were included:

1) patients with glioblastoma treated with temozolomide, annotated with progression-free survival data (n = 49) [32, 33];

2) patients with gastric cancer from a previously published clinical investigation [18] who received ramucirumab as monotherapy (n = 7), or in combination with paclitaxel (n = 6) or the FOLFIRI regimen (n = 2). Response to therapy as well as progression- free survival was assessed;

3) patients with multiple myeloma (n = 60) who received complex chemotherapy in several regimens, each including bortezomib. The response to therapy was registered [34].

**Construction of ERK1/2 molecular pathways and assessment of pathway activation level (PAL) values** The ERK1 (MAPK3) and ERK2 (MAPK1) molecular pathways were algorithmically reconstructed as previously reported in [21]. The human interactome model was constructed using the OncoboxPD collection of published molecular pathways [35] as a molecular interaction database. In total, the architecture of 50 178 distinct molecular pathways was used to build the interactome model. All pathway graphs were merged based on overlapping gene products. The included genes form a connected network, meaning that at least one undirected edge exists between any pair of gene products. As a result, a directed graph was obtained, where nodes represent genes or metabolites, and edges correspond to the known pairwise molecular interactions included in the OncoboxPD collection. The interactome model was visualized using the Gephi software and the ForceAtlas2 algorithm.

For each ERK1 and ERK2 protein, genecentric algorithmic molecular pathways were constructed, including central nodes (ERK1 and ERK2, respectively) and gene products with first-order interactions with the corresponding central nodes. The following types of interactions were considered: “activation”, “coupling”, “inhibition”, “phosphorylation”, “dissociation”, “repression”, “dephosphorylation”, “binding/association”, and “ubiquitination”.

The pathway activation level (PAL) is an aggregate quantitative and qualitative characterization of the changes in the expression level of the genes involved in a particular molecular pathway [36]. The PAL values were calculated as follows:

PALp = 100 × Σ_n_(ARR_n,p_ × lg(CNR_n_))/Σ_n_|ARR_n,p_|,

where PAL*p *is the level of activation of the pathway* p*; CNR*n *is the ratio of the expression of gene *n *in the tested sample to its average level in the control group; and ARR is the role (activator/repressor) played by the given gene product in the *p *pathway. ARR can take on the following values:

-1: when the *n *gene product is a repressor of the *p* pathway;

-0.5: when the *n *gene product is mainly a repressor; 0: when the role of the *n *gene product in the *p *pathway is neutral, uncertain or ambiguous; 0.5: when the *n *gene product is predominantly an activator;

1: when the gene product *n *is an activator.

The ARR values were assigned algorithmically based on the pathway architecture and central node position [14], and PAL calculations were performed using the Python library “oncoboxlib” [14].

If data were downloaded in non-normalized form, normalization of gene expression was performed using DeSeq2 [37]. An artificial gene expression profile obtained by averaging all gene expression data in the study cohort was used as a reference (control) gene expression profile for each individual dataset.

**Statistical tests**

Statistical analyses were performed in R, version 3.4.2 [38]. The PAL or central gene expression level values were divided into groups with a high and low PAL score/gene expression level, depending on whether the score was above or below the optimal value corresponding to the minimum *p-*value of the log-rank test calculated using the “surv_cutpoint” function of the R package “surviminer” [39].

Survival associations were assessed using the Kaplan–Meier method and log-rank test to determine the statistical significance of the difference between the two groups; the hazard ratio (HR, 95% CI) was calculated using the Cox regression model to assess the differences in survival chances between the compared groups using the R packages “survival” [40] and “survminer” [39]. Overall survival (OS) was calculated to the date of death or to the date of the last followup; patients who were alive at the time of last followup were censored. Progression-free survival (PFS) was calculated up to the date of progression, death, or last follow-up. Surviving patients, as well as patients without progression at the date of the last follow-up, were censored. Hazard ratios with *p* < 0.05 and 95% CI not including 1 were considered statistically significant.

In the analysis of the responsiveness to the therapy, when no “responder” nor “non-responder” marks were available in the dataset, the patients with the RECIST *Complete Response *and *Partial Response *outcomes were considered *responders*, and patients with the *Progressive Disease *label were considered *nonresponders*, whereas patients with the *Stable Disease* outcome were considered separately.

Normality of distribution was assessed using the Shapiro–Wilk test; homogeneity of variance, using the Levene’s test. If the number of compared groups exceeded 2, ANOVA or the Kruskal–Wallis test was used depending on whether the distribution met the criteria of normal distribution or not, followed by post-hoc comparison by the Student’s or Dunn’s test, respectively, with correction for multiple comparisons by Benjamini–Hochberg or Holm, respectively. If the number of groups compared was 2, the analyses were performed using the Student’s or Wilcoxon’s test, depending on the normality of distribution. Intergroup comparisons were performed using the R packages “FSA” [41] and “car” [42].

Data visualization was performed using the R packages “ggplot2” [43] and “ComplexHeatmap” [44]. Differences were considered statistically significant at *p* < 0.05.

## RESULTS

**Algorithmic reconstruction of the ERK1 and ERK2 genecentric molecular pathways**

**Fig. 1:**
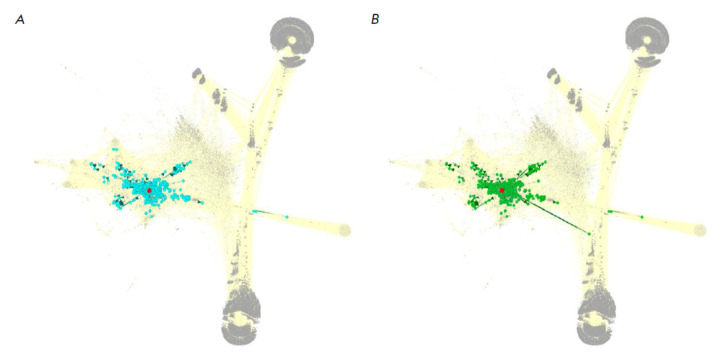
Schematic representation of the composition of algorithmically built molecular pathways centered around the ERK1 (*A*) and ERK2 (*B*) proteins. The gene products participating in the ERK1 and ERK2 signaling pathways are highlighted in the context of the model of human interactome encompassing 361 654 protein–protein interactions across 64 095 molecular players [35]. Red dots represent the central nodes of the pathways (ERK1 or ERK2); projections of pathway members are shown in blue and green for the ERK1 and ERK2 molecular pathways, respectively. Other nodes are shown in grey, with the rest of the interactome graph shown as a background. Visualized using the Gephi software and ForceAtlas2 algorithm [35]

Both pathways were reconstructed based on a previously developed human interactome model represented as a graph comprising 361 654 interactions among 64 095 molecular players. The pathways included members directly interacting with the central node (ERK1/MAPK3 or ERK2/MAPK1, respectively). Annotation of the functional roles of the pathway components was performed algorithmically according to [14]. The resulting pathways (*Fig. 1*) contained 447 and 443 molecular players, respectively. The functionally annotated list of pathway members is provided in *Supplementary Table 1*. A total of 428 members of these pathways (95.7 and 96.6%, respectively) were shared, evidence of their close structural similarity.

**Prognostic significance of the activation of the ERK1/2 pathway and gene expression in relation to cancer patient survival in TCGA data**

RNA sequencing data from the TCGA repository were analyzed to assess the degree of association between patient survival and the expression of the *MAPK3 *and *MAPK1 *genes, as well as the PAL values of the newly reconstructed ERK1 and ERK2 pathways. Our analysis revealed that the PAL values for the ERK1 and ERK2 pathways generally exhibit similar distributions across various tumor types. At that stage, the genecentric KRAS pathway was additionally included in the analysis. this justified by the key role played by RAS family gene products in the activation of the RAS-RAF-MEK-ERK cascade (*Supplementary Fig. 1A*). The KRAS pathway was found to generally display a broader distribution and lower PAL values (except in pheochromocytoma and paraganglioma, where its median value is higher) compared to the ERK1/2 pathways. However, the overall trends in PAL variability across these molecular pathways are consistent (*Supplementary Fig. 1B*). The activation of the KRAS pathway is directly linked to the activation of ERK1/2, providing a means to assess the interplay between these signaling pathways and identify differences in their activity across tumor types. In this case, despite similar trends in PAL values within various tumor types for all three pathways, the lower PAL values observed for the KRAS pathway suggest that ERK pathway activation in these tumors may occur via alternative mechanisms that are independent of KRAS activity.

**Fig. 2:**
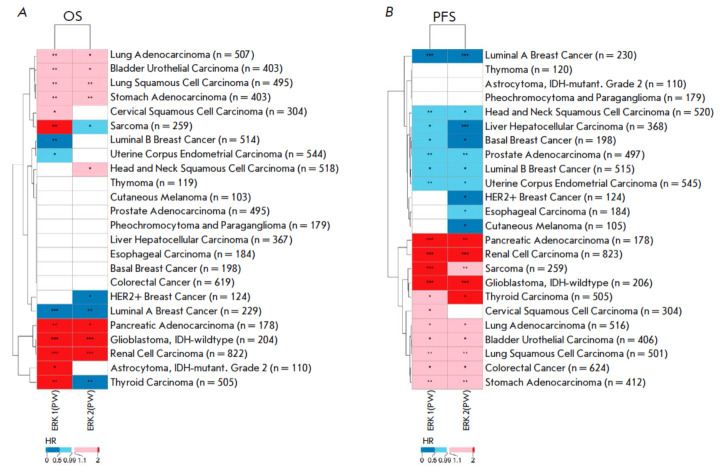
The heatmap of hazard ratio values calculated for the activation of the ERK1 and ERK2 molecular pathways for the (*A*) TCGA overall survival (OS) and (*B*) progression-free survival (PFS) data. HR – hazard ratio; PW – pathway; * *p* < 0.05; ** *p* < 0.01; *** *p* < 0.001

At the next stage, for each cancer type and each putative biomarker, patients were divided into two groups based on whether the PAL or gene expression value was above or below the optimal cut-off point. The Kaplan–Meier method was employed to estimate the chances of survival. The log-rank test was used to assess the statistical significance of the differences between the two groups. In addition, the hazard ratio (HR) and its 95% confidence interval (CI) were calculated. The data were grouped according to the HR value and its statistical significance, and the results were presented as a heatmap with hierarchical clustering (*Fig. 2*).

For the overall survival (OS) data, different cancer types showed differential clustering when grouped according to HR values for the ERK1/2 pathways (*Fig. 2A*). Both pathways generally showed consistent patterns. Specifically, for glioblastoma, kidney, pancreatic, gastric, bladder, lung adenocarcinoma and lung squamous cell carcinoma, activation of both of the ERK1/2 molecular pathways was associated with significantly lower patient OS numbers. Conversely, for the group of genderassociated female tumors (subtypes of breast cancer and endometrioid carcinoma of the uterine corpus), activation of both pathways was a positive prognostic biomarker of OS (*Fig. 2A*). Interestingly, conflicting trends were observed in the prognostic significance of ERK1 and ERK2 pathway activation for sarcoma and thyroid cancer. Given the high similarity between these pathways, differences in prognosis may be attributed to variations in the ARR value, which is also considered when calculating PAL and reflects the functional role of the gene product in the pathway under study (*Supplementary Fig. 2*,* Supplementary Table 1*).

The data obtained for progression-free survival (PFS) generally confirmed the observations obtained for overall survival (*Fig. 2B*). Again, activation of the ERK1/2 pathway was a negative biomarker for glioblastoma, renal, pancreatic, gastric, bladder, lung adenocarcinoma, and squamous cell carcinoma of the lung. In addition, for PFS (not so for OS) it was also a negative biomarker for sarcomas, thyroid cancer, and colorectal cancer. As with OS, activation of the ERK1/2 pathways was a positive biomarker for several subtypes of breast cancer and for endometrioid carcinoma of the uterine corpus. In addition (unlike in OS), it was a positive biomarker for the head and neck, liver, and prostate cancers (*Fig. 2B*).

**Fig. 3:**
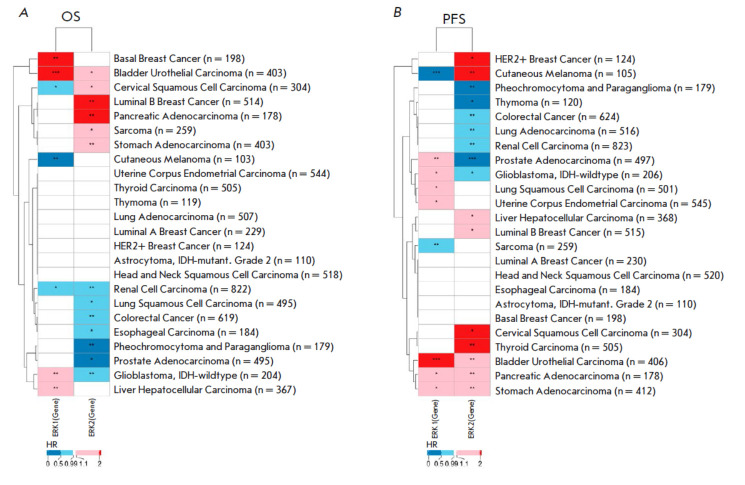
The heatmap of hazard ratio values calculated for the expression levels of the ERK1 and ERK2 individual genes for the (*A*) TCGA overall survival (OS) and (*B*) progression-free survival (PFS) data. HR – hazard ratio; * *p* < 0.05; ** *p* < 0.01; *** *p* < 0.001

In parallel, a similar analysis was performed for the expression levels of the respective central genes of these pathways: *MAPK3* and *MAPK1* (*Fig. 3*). For the individual gene level, only for bladder carcinoma did both genes show a consistent trend (were negative biomarkers) for both OS and PFS data.

**Fig. 4:**
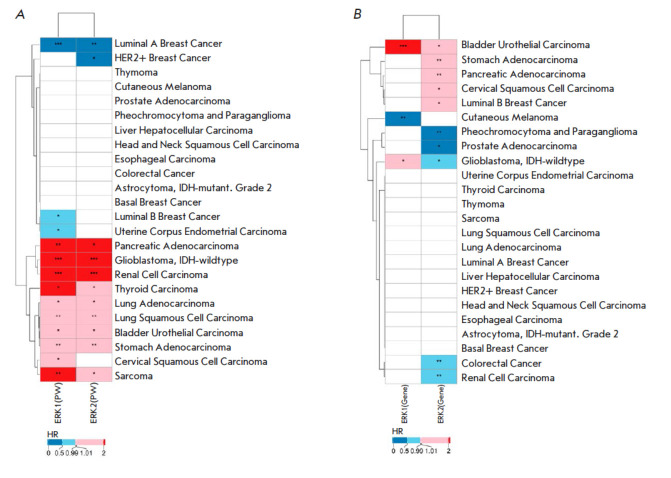
The heatmap of averaged overall survival (OS) and progression-free survival (PFS) hazard ratio values calculated for (*A*) the expression levels of the ERK1 and ERK2 molecular pathways and (*B*) ERK1 and ERK2 individual genes for the TCGA data. HR – hazard ratio; PW – pathway; * *p* < 0.05; ** *p* < 0.01; *** *p* < 0.001. If the orders of significance level of* p*-values differed for the OS and PFS data, the lower significance level was shown

We then averaged the HR values for both OS and PFS data for the PAL and single gene expression types of analyses (*Fig. 4*). For the average HR of PAL, a clear separation of cancer types into two clusters was observed (*Fig. 4A*), whereas the analyses of single gene expression levels showed no definitive clustering (*Fig. 4B*). Hence, the pathway activation analysis returned more consistent and stable results than the assessment of single gene expression levels did. This phenomenon is most probably related to the more stable nature of the pathway-based, aggregated gene expression data, as has been theoretically and experimentally confirmed in previous works [3, 15, 22].

We also calculated the percentage of cancer types where the individual *MAPK3 *and *MAPK1 *genes and respective genecentric molecular pathways could be statistically significant potential prognostic biomarkers according to the TCGA data (*Table 4*). Overall, the activation of molecular pathways was a putative prognostic biomarker more frequently than the expression of the central genes of the respective pathways.

**Table 4:** The percentage of cancer types where the ERK1/2 genes or ERK1/2-centric molecular pathways can be potential prognostic biomarkers in the TCGA data

Type of analysis	Type of biomarker	ERK1, %	ERK2, %
Molecular pathway	Negative	42	42
	Positive	12.5	8
Individual gene	Negative	8	21
	Positive	4	21

**Fig. 5:**
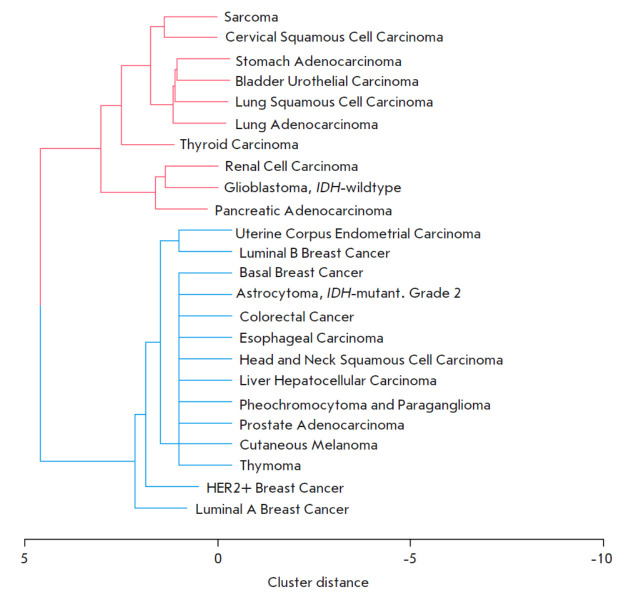
Dendrogram based on clustering tumors by hazard ratio calculated for the ERK1- and ERK2-centric pathway activation data using TCGA gene expression profiles

A dendrogram was then constructed showing the structure of the resulting clusters of cancer types in relation to the HR values calculated for the molecular pathway activation data (*Fig. 5*).

The dendrogram clearly shows two clusters including 10 and 12 cancer types, respectively; within each of those, activation of the ERK1 and ERK2 molecular pathways has similar prognostic value. Thus, the first cluster of 10 cancer types includes the gastric, pancreatic, lung, kidney, bladder, thyroid, cervical, sarcoma, and glioblastoma cancers, where ERK1/2 pathway activation is a rather negative prognostic biomarker (for 90–100% of cluster 1 cancer types). The second cluster includes 12 other tumor types for which activation of these pathways is either a positive prognostic biomarker (HER2+, luminal A and luminal B breast cancer, uterine corpus cancer; a total of 17–25% of cluster 2 cancer types) or has no prognostic value (basal breast cancer, hepatocellular carcinoma, melanoma, etc.; a total of ~75% of cluster 2 cancer types).

**Prognostic significance of ERK1/2 pathway activation according to RNA expression datasets in the literature**

In this study, we assessed the prognostic values of ERK1/2 pathway activation levels and individual genes using an additional set of previously published clinically annotated gene expression profiles collected by Borisov et al. [28]. Based on the data in the literature, patient responses to therapy were evaluated according to the RECIST criteria [27].

Our analysis yielded statistically significant differences in the PAL values between response groups for patients with the following cancers: colorectal cancer, sarcomas, breast cancer, lung adenocarcinoma, and multiple myeloma.

**Fig. 6:**
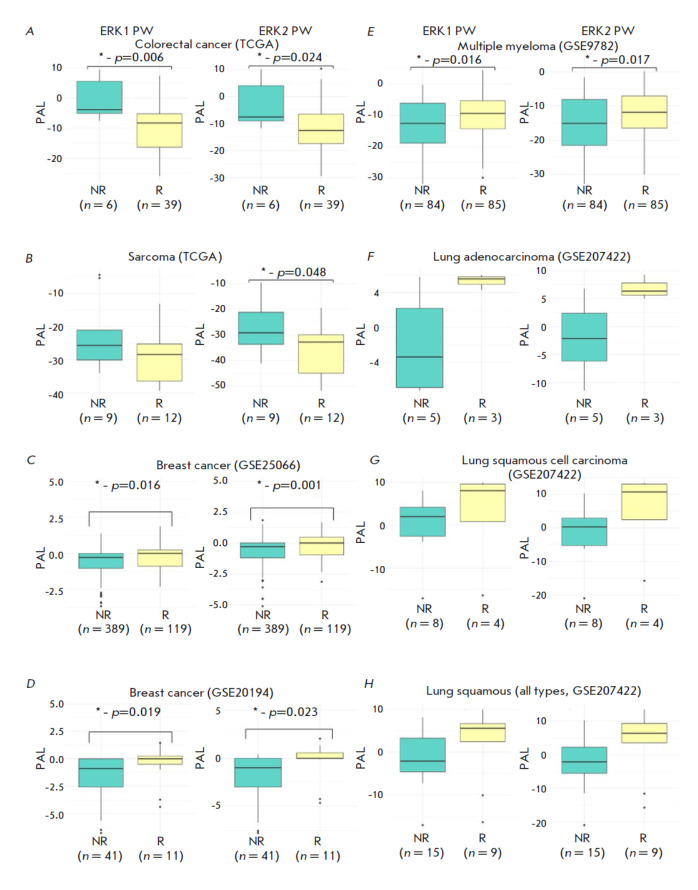
Differences in PAL of the studied molecular pathways according to the response to therapy (*A*) with a combination of 5-fluorouracil, leucovorin, and oxaliplatin in patients with colorectal cancer from the TCGA project; (*B*) with a combination of docetaxel and gemcitabine in sarcoma patients from the TCGA project; (*C*) with taxane and anthracycline in breast cancer patients from the GEO_2_5066 dataset; (*D*) with taxane and anthracycline in breast cancer patients from the GEO_2_0194 dataset; (*E*) with bortezomib in multiple myeloma patients from the GSE9782 dataset; (*F*) with immunotherapy in patients with lung adenocarcinoma from the GSE207422 dataset; (*G*) immunotherapy in patients with lung squamous cell carcinoma from the GSE207422 dataset; (*H*) immunotherapy in patients with lung cancer (all histological types) from the GSE207422 dataset. The results of the RECIST response analysis are presented as boxplots, where the horizontal line represents the median; the first and third quartiles are represented by the lower and upper boundaries of the rectangle; the minimum and maximum observed values are indicated by the ends of the vertical lines; and possible outliers are shown as individual points beyond them. Statistically significant differences are marked with * , indicating the exact *p*-value. “R” stands for treatment responders; “NR”, for non-responders, and “PW”, for pathway

Interestingly, the results of the analysis of the response to therapy for patients with colorectal cancer (*Fig. 6A*) and sarcomas (*Fig. 6B*) from the TCGA project are consistent with the results of the HR analysis and the prognostic value of the molecular pathways studied for the survival of patients from the same TCGA datasets in the larger sample. In both cancer types, patients with lower activation of one or both of the studied molecular pathways responded better to therapy.

In turn, the previously reported positive prognostic significance of ERK1/2 pathway activation in breast cancer was confirmed in two gene expression datasets for patients receiving combination treatment with taxanes and anthracyclines: GSE25066 (paclitaxel, 5-fluorouracil, cyclophosphamide, doxorubicin or epirubicin in adjuvant and neoadjuvant regimens) and GSE20194 (paclitaxel, 5-fluorouracil, cyclophosphamide, doxorubicin in combination in neoadjuvant regimen), (*Fig. 6C,D*).

In addition, in this study, we evaluated the association between PAL and the response to therapy in blood tumors. Statistically significant relationships were identified for the multiple myeloma dataset in patients receiving bortezomib monotherapy (GSE9782). Although this cancer type was not included in the previous TCGA analysis, the results suggest that activation of the ERK1/2 pathway may point to a positive survival prognosis (*Fig. 6E*).

Furthermore, the association between the ERK1/2 pathway PAL and the response to anti-PD1 immunotherapy was evaluated in patients with lung cancer using the GSE207422 dataset. This dataset included RNA expression data from 24 lung cancer patients, of whom twelve were diagnosed with squamous cell lung cancer, eight, with lung adenocarcinoma, and the remaining patients had other diagnoses. For both the entire sample and the individual squamous cell lung cancer and lung adenocarcinoma groups, there was a trend for patients who responded to immunotherapy to demonstrate higher ERK1/2 activation in tumor samples (*Fig. 6F-H*). However, because the sample was small and the observed difference did not reach the level of statistical significance, this finding needs to be revisited in an independent analysis using a larger cohort of patients.

In summary, an analysis of clinical datasets confirmed that activation of ERK1/2 molecular pathways may be closely associated with the response to several anticancer therapies, such as in breast cancer, colorectal cancer, and sarcomas.

**Prognostic significance of ERK1/2 pathway activation according to the original experimental RNA expression datasets**

The previous findings were supplemented with the results obtained using our proprietary gene expression datasets previously published by our team for cancer patients annotated with the therapy response. Three of our previous clinical datasets were considered here: glioblastoma (*n *= 49), stomach cancer (*n *= 15), and multiple myeloma (*n *= 60) patients receiving anticancer therapy. The response to the therapy was assessed either according to the PFS alone (glioblastoma), the RECIST criteria alone (multiple myeloma), or both the PFS and the RECIST criteria (gastric cancer).

**Fig. 7:**
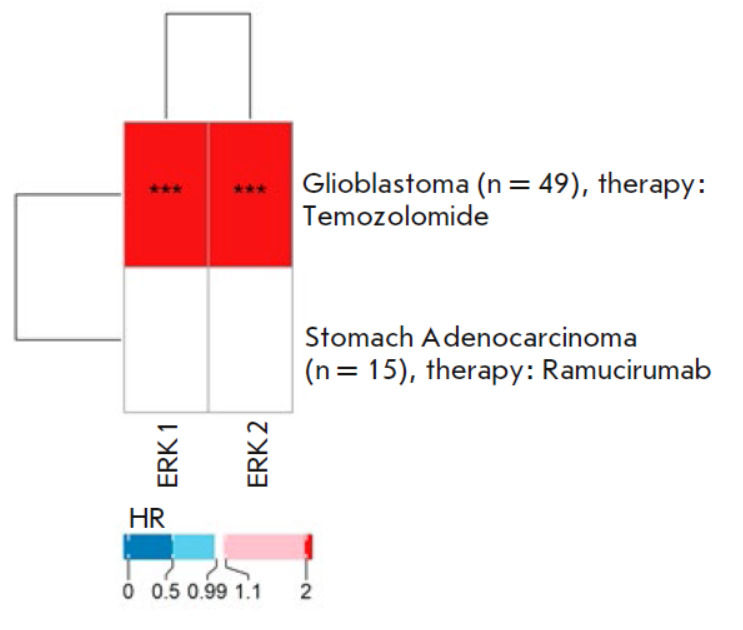
The heatmap of the hazard ratio values calculated for the activation of the molecular pathways ERK1 and ERK2 based on pathway activation levels in relation to the response to anticancer chemotherapy, as assessed by progression-free survival (PFS). HR – hazard ratio; *** *p* < 0.001

The PFS data results revealed a strongly negative prognosis for ERK1/2 activation in response to the temozolomide therapy in glioblastoma and no significant association for the treatment of stomach cancer patients with the targeted therapeutic ramucirumab (*Fig. 7*). No statistically significant difference could be observed for the ERK1/2 pathway activation for the RECIST responder and non-responder patient data.

In the multiple myeloma dataset, we also found no statistically significant difference in ERK1/2 PAL values for patients who responded or did not respond well to treatment with bortezomib-containing regimens.

## DISCUSSION

In this study, we for the first time algorithmically reconstructed molecular pathways for the regulatory protein kinases ERK1 and ERK2 using a whole intractome model. We then examined the relationship between the activation levels of these pathways and the available data on patient survival and sensitivity to different therapeutic regimens in different cancer types.

The results suggest that cancer types can be divided into three classes, in which ERK1/2 pathway activation may be either a negative or positive prognostic biomarker or may not be statistically significant at all. Specifically, the first class of such cancers includes gastric cancer, two different histologic types of lung cancer, glioblastoma, sarcomas, kidney cancer, and some other cancers (*Fig. 4*). Our results are also consistent with the literature: for example, an experimental association between ERK activation and a negative prognosis is known for gastric cancer [45, 46], kidney, bladder and lung adenocarcinoma [47]. For glioblastomas, angiogenic factors and receptors were shown to play one of the key roles in their development; in particular, activate the RAS-RAF-MEK-ERK axis and promote the proliferation, migration, and survival of malignant cells [48]. In our study, activation of the ERK pathway was associated with shorter PFS for glioblastoma patients after therapy with the alkylating drug temozolomide. Therefore, activation of the ERK1/2-pathway in glioblastoma may potentially be not only a prognostic biomarker of survival, but also a biomarker of the response to this type of therapy.

The second class includes cancers for which activation of the ERK1/2 pathway was a positive prognostic biomarker (HER2+, luminal A and luminal B breast and uterine corpus cancers). ERK1 activation has previously been shown to be associated with a better prognosis for breast cancer patients, because it leads to the blockage of the Hippo signaling pathway and one of its downstream targets, the YAP1 protein. However, in the same study, ERK2 activation proved to be associated with a negative prognosis [49]. On the other hand, it has recently been shown that HER2+ breast cancer is resistant to targeted therapy when ERK1/2 kinase activity is low, and that high kinase activity is a prognostic biomarker of tumor sensitivity to therapy [50]. It is consistent with our results, where positive associations were also shown for breast cancer sensitivity to taxanes and anthracyclines, whereas expression of the individual corresponding central genes of these pathways was a much less accurate biomarker.

Finally, for the third class, which includes basal breast cancer, hepatocellular carcinoma, melanoma, and some other cancers, no significant biomarker potential could be detected for activation of the ERK1/2 pathway. We believe that, taken together, these results may be useful for cataloging clinically relevant alterations in intracellular signaling in cancers, and for further developing combination cancer therapies that may include targeted ERK1/2 inhibitors. It may also be useful to establish adequate models for testing such drug combinations, since activation of the ERK1/2 pathway may have opposite effects on the therapeutic success of treatments for different types of human cancers.

## CONCLUSION

In this study, we showed that the level of activation of algorithmically reconstructed ERK1/2 signaling pathways may be an effective prognostic and predictive cancer biomarker, with its prognostic value and significance depending strongly on cancer type and the type of therapy.

## Abbreviations

MAPK: mitogen-activated protein kinase
PAL: pathway activation level
CNR: case-tonormal ratio
ARR: activation/repressor role
OS: overall survival
PFS: progression-free survival
HR: hazard ratio
